# Supplementary material for: Modeling glioblastoma heterogeneity as a dynamic network of cell states
Source: Mol Syst Biol. 2021 Sep 16;17(9):e10105. doi: 10.15252/msb.202010105 (PMC8444284; doi:10.15252/msb.202010105)
Supplement: Supplementary file 6 — Source Data for Figure 5 [file MSB-17-e10105-s004.zip › Figure5A_sourcedata/GSEA_3071/nosigdb_stateII.GseaPreranked.1622795151171/FELDMAN_2020_S.html]

Details for gene set FELDMAN\_2020\_S[GSEA]

|  || Dataset | state23071 |
| Phenotype | NoPhenotypeAvailable |
| Upregulated in class | na\_pos |
| GeneSet | FELDMAN\_2020\_S |
| Enrichment Score (ES) | 0.6647973 |
| Normalized Enrichment Score (NES) | 4.254443 |
| Nominal p-value | 0.0 |
| FDR q-value | 0.0 |
| FWER p-Value | 0.0 |
Table: GSEA Results Summary

  

Fig 1: Enrichment plot: FELDMAN\_2020\_S      
 Profile of the Running ES Score & Positions of GeneSet Members on the Rank Ordered List

  

| PROBE | GENE SYMBOL | GENE\_TITLE | RANK IN GENE LIST | RANK METRIC SCORE | RUNNING ES | CORE ENRICHMENT || 1 | CLSPN |  |  | 1 | 1.984 | 0.0164 | Yes |
| 2 | RRM2 |  |  | 3 | 1.857 | 0.0317 | Yes |
| 3 | TYMS |  |  | 8 | 1.665 | 0.0446 | Yes |
| 4 | MCM10 |  |  | 9 | 1.654 | 0.0584 | Yes |
| 5 | CDC6 |  |  | 11 | 1.631 | 0.0718 | Yes |
| 6 | CDK1 |  |  | 14 | 1.540 | 0.0842 | Yes |
| 7 | ZWINT |  |  | 16 | 1.510 | 0.0966 | Yes |
| 8 | ATAD5 |  |  | 18 | 1.469 | 0.1087 | Yes |
| 9 | FEN1 |  |  | 19 | 1.464 | 0.1209 | Yes |
| 10 | BRCA2 |  |  | 22 | 1.445 | 0.1325 | Yes |
| 11 | CENPU |  |  | 24 | 1.416 | 0.1441 | Yes |
| 12 | CENPK |  |  | 26 | 1.382 | 0.1554 | Yes |
| 13 | CDCA4 |  |  | 27 | 1.376 | 0.1670 | Yes |
| 14 | FAM111B |  |  | 29 | 1.361 | 0.1781 | Yes |
| 15 | FBXO5 |  |  | 30 | 1.359 | 0.1895 | Yes |
| 16 | USP1 |  |  | 31 | 1.357 | 0.2009 | Yes |
| 17 | PSMC3IP |  |  | 36 | 1.322 | 0.2109 | Yes |
| 18 | ORC6 |  |  | 39 | 1.308 | 0.2214 | Yes |
| 19 | GINS2 |  |  | 40 | 1.306 | 0.2323 | Yes |
| 20 | TK1 |  |  | 42 | 1.281 | 0.2428 | Yes |
| 21 | TAGLN |  |  | 43 | 1.279 | 0.2535 | Yes |
| 22 | MCM7 |  |  | 45 | 1.273 | 0.2639 | Yes |
| 23 | HELLS |  |  | 46 | 1.259 | 0.2744 | Yes |
| 24 | BRCA1 |  |  | 49 | 1.249 | 0.2844 | Yes |
| 25 | DNAJC9 |  |  | 56 | 1.217 | 0.2931 | Yes |
| 26 | PCNA |  |  | 58 | 1.214 | 0.3030 | Yes |
| 27 | CDC45 |  |  | 59 | 1.212 | 0.3131 | Yes |
| 28 | MAD2L1 |  |  | 61 | 1.199 | 0.3229 | Yes |
| 29 | UBE2T |  |  | 62 | 1.192 | 0.3329 | Yes |
| 30 | ATAD2 |  |  | 63 | 1.189 | 0.3429 | Yes |
| 31 | MSH2 |  |  | 66 | 1.163 | 0.3521 | Yes |
| 32 | RFC3 |  |  | 67 | 1.157 | 0.3618 | Yes |
| 33 | POLD3 |  |  | 70 | 1.143 | 0.3708 | Yes |
| 34 | MCM5 |  |  | 74 | 1.128 | 0.3795 | Yes |
| 35 | CCNE2 |  |  | 76 | 1.121 | 0.3887 | Yes |
| 36 | MCM4 |  |  | 81 | 1.110 | 0.3969 | Yes |
| 37 | GMNN |  |  | 83 | 1.108 | 0.4060 | Yes |
| 38 | VRK1 |  |  | 93 | 1.074 | 0.4127 | Yes |
| 39 | DHFR |  |  | 101 | 1.051 | 0.4197 | Yes |
| 40 | MCM3 |  |  | 105 | 1.035 | 0.4276 | Yes |
| 41 | RFC2 |  |  | 112 | 1.022 | 0.4347 | Yes |
| 42 | DUT |  |  | 118 | 1.009 | 0.4419 | Yes |
| 43 | ASF1B |  |  | 120 | 1.007 | 0.4501 | Yes |
| 44 | SMC2 |  |  | 123 | 1.005 | 0.4580 | Yes |
| 45 | CHAF1A |  |  | 127 | 0.999 | 0.4656 | Yes |
| 46 | ESCO2 |  |  | 144 | 0.971 | 0.4697 | Yes |
| 47 | RANBP1 |  |  | 149 | 0.958 | 0.4767 | Yes |
| 48 | GGCT |  |  | 160 | 0.937 | 0.4820 | Yes |
| 49 | RPA2 |  |  | 169 | 0.929 | 0.4878 | Yes |
| 50 | MCM6 |  |  | 176 | 0.906 | 0.4938 | Yes |
| 51 | CENPM |  |  | 178 | 0.901 | 0.5011 | Yes |
| 52 | TFDP1 |  |  | 209 | 0.866 | 0.5008 | Yes |
| 53 | H2AFZ |  |  | 213 | 0.864 | 0.5073 | Yes |
| 54 | CDCA3 |  |  | 219 | 0.858 | 0.5132 | Yes |
| 55 | MIS18A |  |  | 239 | 0.833 | 0.5154 | Yes |
| 56 | C19orf48 |  |  | 248 | 0.826 | 0.5203 | Yes |
| 57 | LMNB1 |  |  | 251 | 0.824 | 0.5267 | Yes |
| 58 | TPM1 |  |  | 259 | 0.805 | 0.5317 | Yes |
| 59 | DNMT1 |  |  | 265 | 0.792 | 0.5370 | Yes |
| 60 | YWHAH |  |  | 273 | 0.781 | 0.5418 | Yes |
| 61 | HMGB2 |  |  | 276 | 0.780 | 0.5478 | Yes |
| 62 | EIF2S1 |  |  | 282 | 0.776 | 0.5531 | Yes |
| 63 | HSPB11 |  |  | 283 | 0.775 | 0.5596 | Yes |
| 64 | TEX30 |  |  | 287 | 0.774 | 0.5653 | Yes |
| 65 | DEK |  |  | 302 | 0.754 | 0.5681 | Yes |
| 66 | CENPA |  |  | 303 | 0.751 | 0.5744 | Yes |
| 67 | SLBP |  |  | 318 | 0.740 | 0.5770 | Yes |
| 68 | ANP32B |  |  | 325 | 0.734 | 0.5816 | Yes |
| 69 | PHLDA2 |  |  | 332 | 0.726 | 0.5862 | Yes |
| 70 | AKAP12 |  |  | 349 | 0.711 | 0.5881 | Yes |
| 71 | AURKA |  |  | 360 | 0.705 | 0.5915 | Yes |
| 72 | SIVA1 |  |  | 380 | 0.690 | 0.5925 | Yes |
| 73 | RFC4 |  |  | 385 | 0.686 | 0.5972 | Yes |
| 74 | EXOSC9 |  |  | 399 | 0.679 | 0.5996 | Yes |
| 75 | TMEM97 |  |  | 403 | 0.676 | 0.6045 | Yes |
| 76 | CTNNAL1 |  |  | 405 | 0.676 | 0.6099 | Yes |
| 77 | CCDC34 |  |  | 411 | 0.672 | 0.6143 | Yes |
| 78 | PA2G4 |  |  | 412 | 0.672 | 0.6199 | Yes |
| 79 | RAN |  |  | 424 | 0.667 | 0.6227 | Yes |
| 80 | SMC3 |  |  | 425 | 0.667 | 0.6283 | Yes |
| 81 | HNRNPAB |  |  | 435 | 0.659 | 0.6316 | Yes |
| 82 | ACAT2 |  |  | 446 | 0.653 | 0.6345 | Yes |
| 83 | SNRPB |  |  | 467 | 0.642 | 0.6348 | Yes |
| 84 | DCTPP1 |  |  | 477 | 0.638 | 0.6379 | Yes |
| 85 | PGP |  |  | 492 | 0.631 | 0.6397 | Yes |
| 86 | RAD51 |  |  | 506 | 0.627 | 0.6416 | Yes |
| 87 | SNRPD1 |  |  | 523 | 0.615 | 0.6428 | Yes |
| 88 | HAUS8 |  |  | 527 | 0.613 | 0.6471 | Yes |
| 89 | RPA3 |  |  | 532 | 0.611 | 0.6512 | Yes |
| 90 | HSPA8 |  |  | 547 | 0.603 | 0.6528 | Yes |
| 91 | MEST |  |  | 563 | 0.593 | 0.6539 | Yes |
| 92 | EXOSC8 |  |  | 604 | 0.576 | 0.6487 | Yes |
| 93 | MSH6 |  |  | 611 | 0.574 | 0.6520 | Yes |
| 94 | CACYBP |  |  | 613 | 0.573 | 0.6565 | Yes |
| 95 | HAT1 |  |  | 632 | 0.565 | 0.6567 | Yes |
| 96 | NCL |  |  | 638 | 0.560 | 0.6601 | Yes |
| 97 | PPIF |  |  | 639 | 0.560 | 0.6648 | Yes |
| 98 | DLGAP5 |  |  | 712 | 0.529 | 0.6511 | No |
| 99 | NASP |  |  | 741 | 0.518 | 0.6483 | No |
| 100 | UNG |  |  | 750 | 0.516 | 0.6506 | No |
| 101 | EIF4EBP1 |  |  | 805 | 0.496 | 0.6412 | No |
| 102 | EEF1E1 |  |  | 840 | 0.488 | 0.6367 | No |
| 103 | ARL6IP6 |  |  | 880 | 0.476 | 0.6308 | No |
| 104 | DERA |  |  | 886 | 0.474 | 0.6335 | No |
| 105 | SRSF2 |  |  | 941 | 0.461 | 0.6238 | No |
| 106 | LIMS1 |  |  | 951 | 0.458 | 0.6253 | No |
| 107 | SRSF7 |  |  | 1071 | 0.424 | 0.5988 | No |
| 108 | HNRNPF |  |  | 1128 | 0.412 | 0.5882 | No |
| 109 | POLR3K |  |  | 1140 | 0.410 | 0.5888 | No |
| 110 | EIF1AX |  |  | 1198 | 0.397 | 0.5778 | No |
| 111 | PTMA |  |  | 1233 | 0.390 | 0.5724 | No |
| 112 | HES4 |  |  | 1250 | 0.387 | 0.5716 | No |
| 113 | RMI2 |  |  | 1279 | 0.381 | 0.5678 | No |
| 114 | MAGOHB |  |  | 1336 | 0.369 | 0.5567 | No |
| 115 | RNASEH2A |  |  | 1341 | 0.369 | 0.5588 | No |
| 116 | SLC20A1 |  |  | 1400 | 0.357 | 0.5472 | No |
| 117 | CCND1 |  |  | 1753 | 0.304 | 0.4609 | No |
| 118 | RPL22L1 |  |  | 1966 | 0.277 | 0.4097 | No |
| 119 | RAB13 |  |  | 2381 | -0.268 | 0.3075 | No |
| 120 | MARCKS |  |  | 3369 | -0.487 | 0.0625 | No |
| 121 | ARL6IP5 |  |  | 3529 | -0.557 | 0.0271 | No |
| 122 | ARL6IP1 |  |  | 3574 | -0.586 | 0.0209 | No |
| 123 | YPEL5 |  |  | 3603 | -0.605 | 0.0189 | No |
| 124 | PHLDA1 |  |  | 3622 | -0.617 | 0.0195 | No |
| 125 | CDCA7L |  |  | 3642 | -0.637 | 0.0200 | No |
| 126 | SAT1 |  |  | 3659 | -0.653 | 0.0215 | No |
| 127 | TGIF1 |  |  | 3728 | -0.707 | 0.0102 | No |
| 128 | BEX4 |  |  | 3796 | -0.785 | -0.0001 | No |
| 129 | SOX4 |  |  | 3797 | -0.786 | 0.0065 | No |
| 130 | PAIP2 |  |  | 3830 | -0.821 | 0.0053 | No |
| 131 | LYPD1 |  |  | 3844 | -0.834 | 0.0090 | No |
| 132 | TSC22D1 |  |  | 3872 | -0.887 | 0.0096 | No |
| 133 | ZFAND6 |  |  | 3904 | -0.952 | 0.0097 | No |
| 134 | SESN3 |  |  | 4003 | -1.260 | -0.0044 | No |
| 135 | NOVA1 |  |  | 4008 | -1.312 | 0.0055 | No |
| 136 | SCRG1 |  |  | 4070 | -2.021 | 0.0071 | No |
Table: GSEA details [plain text format]

  

Fig 2: FELDMAN\_2020\_S: Random ES distribution      
 Gene set null distribution of ES for **FELDMAN\_2020\_S**
